# Medical Institute of Louisville

**Published:** 1841-01

**Authors:** 


### MEDICAL INSTITUTE OF LOUISVILLE.

The annual catalogue of this institution has just been published, from which it appears that the number of its pupils is 205; of whom 70 are from Kentucky, 50 from Tennessee, 21 from Alabama, 19 from Indiana, and 17 from Mississippi; while the remainder are derived from nine other States and Territories, with one from Texas. The Institute opened, in 1837, with a class of 80 students; its second class numbered 120, and its third, 204. Of the twenty six medical schools in the United States, its class, last winter, was the third in number. We have not yet received catalogues from any of the schools, this season Y.

